# The social versus food preference test: A behavioral paradigm for studying competing motivated behaviors in rodents

**DOI:** 10.1016/j.mex.2020.101119

**Published:** 2020-10-29

**Authors:** Christina J. Reppucci, Alexa H. Veenema

**Affiliations:** Department of Psychology & Neuroscience Program, Michigan State University, United States

**Keywords:** Investigation, Social interaction-seeking, Food-seeking, Three-chamber test

## Abstract

Behavior is influenced by a combination of factors, with the expression of the appropriate behavior dependent on an individual's current motivational state and the presence of stimuli in their surrounding environment. Thus far, most laboratory studies have focused on uncovering the peripheral and central systems that regulate the expression of a single behavior or the expression of a suite of behaviors associated with a single motivational state. In natural settings, however, an individual can be simultaneously experiencing multiple motivational states with multiple choices of how to act. Yet, the direct assessment of the roles of peripheral and central systems in coordinating motivated behavioral choice is largely understudied. This may be due to a lack of behavioral tests that are suitable for such investigations. Here, we describe a recently developed behavioral paradigm, hereafter called the Social versus Food Preference Test. This behavioral paradigm was validated in both rats and mice and is highly flexible, which will allow addressing of a wide range of research questions concerning the peripheral and central systems that coordinate the choice to seek social interaction versus the choice to seek food.

• This paradigm was validated in rats and mice, the two most commonly used nonhuman species in behavioral research, but could be adapted for use in other rodent models.
• The specific social and food stimuli used can be selected based on the research question.
• Three-chamber apparatuses can be custom-constructed.

## Graphical abstract

**Figure:**
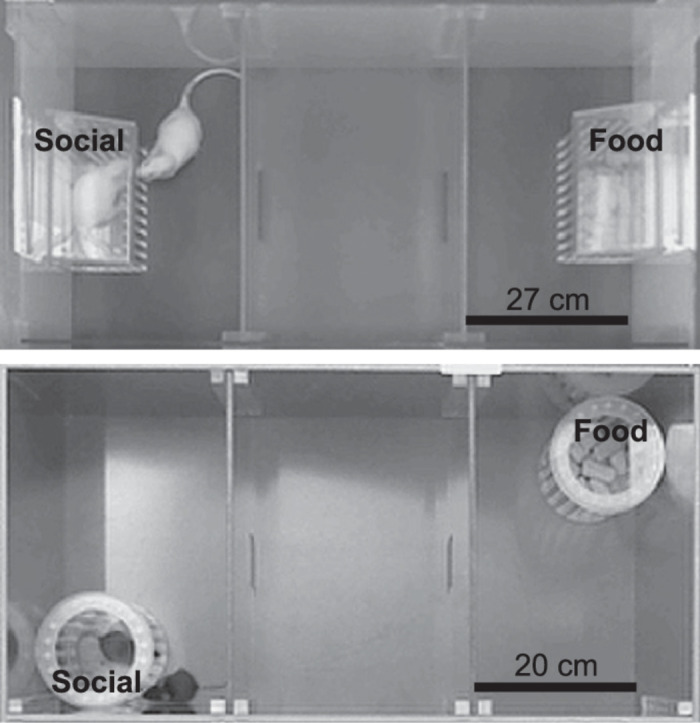

Specifications table

**Table:** 

Subject Area	Neuroscience
More specific subject area	Behavioral Neuroscience; Animal Behavior
Method name	Social versus Food Preference Test
Name and reference of original method	Social interaction assay from: Burnett, C. J., Li, C., Webber, E., Tsaousidou, E., Xue, S. Y., Brüning, J. C., & Krashes, M. J. (2016). Hunger-driven motivational state competition. Neuron, 92(1), 187–201. doi: 10.1016/j.neuron.2016.08.032.
Resource availability	*n/a*

## Method details

### Background

**Fig. 1:**
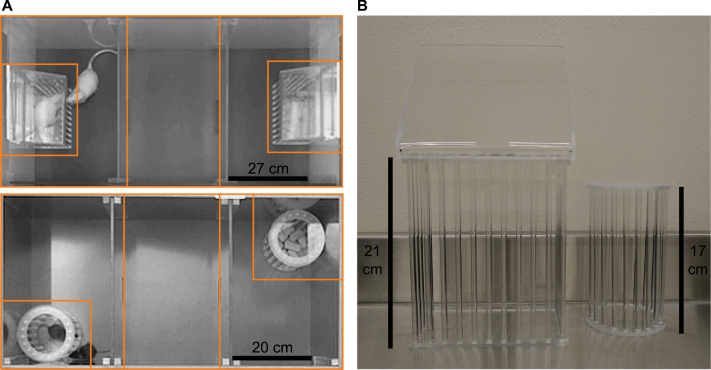
Rats (A, top) and mice (**A**, bottom) are placed into the center of a three-chamber apparatus and then allowed to freely investigate a social stimulus and a food stimulus, which are each placed in corrals located on opposite ends (**B**, rats: left, mice: right), for a period of 10 min. Orange lines in **A** indicate chambers and investigation zones defined in AnyMaze for automated behavioral tracking (middle chamber borders were drawn on the apparatus floor).

The Social versus Food Preference Test was developed to assess the preference of rats and mice to investigate a social stimulus versus a food stimulus, and was based on a two-chamber social interaction assay used to determine the effects of hunger signals on social interest in mice [1]. In our adaptation of this paradigm, we examined social versus food preference using a three-chamber apparatus where the social stimulus and the food stimulus were placed on opposite ends (Fig. 1) [2]. This configuration allows for a neutral middle chamber zone instead of a forced choice that two-chamber configurations elicit, and its use was based on our previous experiences with social novelty preference [3] and opposite sex preference [4] tests in rats, and well-characterized sociability and social novelty preference tests in mice [5].

### Apparatus

Two sizes of the three-chamber apparatus were custom-constructed (Figs. 1A and 8), one for rats (Scientific Instrumental and Machining Services, Boston College) and one for mice (Physics and Astronomy Machine Shop, Michigan State University). The exterior of the apparatus was composed of acrylic (rats) or PVC (mice), and each chamber (rats: 40 cm × 40 cm × 27 cm; mice: 30 cm × 30 cm × 20 cm) was separated by a translucent acrylic partition with an opening (rats: 10 cm × 10.2 cm; mice: 5 cm × 5 cm) to allow passage between chambers. When possible, the walls of the apparatus should be constructed of opaque materials to reduce visual distractions during testing. Alternatively, paint or opaque Con-Tact paper can be applied to the exterior walls. If automated tracking software will be used, the color of the apparatus should contrast to the color of the test subjects. Commercially available three-chamber apparatuses would also be well-suited for this test.

Custom-constructed corrals were used to hold the social and food stimuli (Fig. 1B). For rats, rectangular corrals (18 cm W × 10 cm D × 21 cm H) were composed of a solid translucent acrylic top/bottom/back and translucent acrylic bars (0.6 cm diameter, spaced 1.75 cm apart center-to-center) on the other three sides. For mice, cylindrical corrals (8.5 cm ID, 10.5 cm OD × 17 cm H) were composed of solid translucent acrylic top/bottom connected by translucent acrylic bars (0.6 cm diameter, spaced 1.5 cm apart center-to-center). These corrals allow for olfactory, visual, and auditory contact, but restricted tactile contact of the stimuli by the experimental subject. With these corrals minimal nose or forepaw contact between the experimental and stimulus animals was possible and the experimental animals could sniff the tail of the stimulus animal if it extended outside of the corral, but no anogenital investigation, playful behaviors, or aggressive behaviors were observed. For suggested modifications to these corral specifications, see **Additional Information: Adjustments to reduce climbing of the corrals and/or escape from the apparatus**. Commercially available corrals or pencil cup holders (for mice only) would also be well-suited for this test.

The apparatus should be wiped down with 70% ethanol and corrals should be wiped down with a dilute cleaning solution at the start and end of each day, as well as between subjects.

## Procedure

### Habituation

Experimental subjects and stimulus animals should be habituated to the testing procedures 1–2 days prior to their first test in order to acclimate them to the procedures and apparatus. Experimental subjects are placed into the center chamber and allowed to freely explore the apparatus and investigate empty corrals located on opposite ends for 10 min before being returned to their homecage. Habituation of experimental subjects should be video recorded and the time spent in each chamber measured (see **Behavioral**s **coring**, below). While individual subjects may spend more time in one chamber than the other chambers, as a cohort there should be no difference in the time spent in the two end chambers of the three-chamber apparatus. In separate trials, stimulus animals are habituated to confinement within a corral for 10 min before being returned to their homecage.

### Behavioral testing

1. To reduce the amount of food-related sensory cues present on the social stimuli, remove food from the cages of stimulus animals 2 h prior to the start of testing [1].
2. If testing occurs in a room separate from the housing room, move subjects to the test room at least 1 hr before the test to allow them to acclimatize.
3. Clean apparatus and corrals.
4. Set-up camera for video recording of the tests.
  a. Direct overhead placement is ideal for subsequent videos analyses.
  b. Make sure the apparatus is equally illuminated, and glare minimized.
    i. Overhead white light should be used for testing during the light phase.
    ii. Infrared illuminators and indirect dim red light should be used for testing during the dark phase.
  c. A camera that is connected to a computer in an adjoining room or outside the test room is recommended, so that tests can be monitored remotely and the animals experience no disturbance from the experimenter.
5. Place the selected social stimulus into one corral and the selected food stimulus into a second corral, then put the two corrals on opposite ends of the three-chamber apparatus.
  a. Corrals should be put into a designated location within each chamber (e.g., middle of back wall or back corner).
  b. Because the Social versus Food Preference Test assesses real-time place preference, the location of the social and food stimuli (i.e., left chamber or right chamber) is independent of chamber preference during habituation.
  c. However, the location of the social and food stimuli should be counterbalanced between subjects each test day.
  d. When applicable, the location of the social and food stimuli should also be counterbalanced within subjects across test days to prevent the development of a conditioned place preference.
  e. The specific social and food stimuli should be selected based on the research question.
    i. Potential considerations for the social stimulus: novelty/familiarity, age, sex.
    ii. Potential considerations for the food stimulus: novelty/familiarity, palatability, previously devalued.
  f. Food should be moved away from the accessible edges of the corral to prevent consumption.
6. Start the video recording, then place the experimental subject into the center chamber and allow free exploration of the apparatus for 10 min.
  a. The experimenter should monitor the test, but remain out-of-sight, preferably outside the test room to minimize any disturbances to the animals.
  b. If an experimental subject climbs up and sits on top of the corral or the edge of the apparatus, allow ~10 s for the subject to climb or jump down on their own. If they do not, gently pick up and place the subject on the floor of the chamber they climbed out of (not the center chamber).
7. Stop the video recording, then remove the experimental subject from the apparatus and return it to its homecage.
8. Remove the social stimulus from the corral and return it to its homecage.
9. Remove the food stimulus from the corral and discard.
10. Clean the apparatus and corrals, and reset (starting at item 5) for the next experimental subject (if applicable).

### Behavioral scoring

1. Automated tracking software can be used to quantify a wide range of behaviors.
  a. At a minimum, it is recommended to quantify the time spent in each of the three chambers, and the time spent in a designated “investigation zone” around each of the two corrals (e.g., rats: head placement within 6 cm of corral edge, mice: center mass placement within 5 cm of corral edge; Fig. 1A).
  b. If issues arise with the automated tracking, it could be that there is too much glare, the camera is not positioned directly overhead the apparatus, the software is unable to distinguish between the experiment subject and the social stimulus, the experimental subject climbs the corrals, or the experimental subject does not contrast well enough against the apparatus.
2. Manual scoring can be used instead of, or in addition to, automated tracking software.
  a. At a minimum, it is recommended to quantify the time spent in each of the three chambers (especially if automated tracking is unavailable or technical difficulties arise), as well as the time experimental subjects spent actively investigating each of the two stimuli. Investigation is defined as when the experimental subject's attention is directed towards the stimulus inside of the corral as indicated by head position/gaze orientation, and the subject is engaged with the corral (e.g., sticking nose between bars, pawing, sniffing).
  b. To reduce potential bias, experimenters scoring videos should be unaware of the characteristics and/or test conditions for experimental subjects (e.g., age, sex, homeostatic manipulation, drug condition) and stimuli (e.g., familiar, novel). However, blinding to the stimulus category is difficult and should be avoided if possible, since investigation directed towards the social and food stimuli is qualitatively different.
    i. For the food stimulus, investigation is almost exclusively around the bottom portion of the corral. Rearing and sniffing the top portion of the corral is not scored as investigation since the experimental subject's attention is not directed at the food placed on the floor of the corral.
    ii. For the social stimulus, rearing and sniffing the top portion of the corral by the experimental subject can be scored as investigation if the stimulus animal is also rearing and it is clear that the experimental subject's attention is directed towards the social stimulus inside.
3. Data can be analyzed to assess both the absolute (i.e., time in seconds) and the relative (i.e., preference) interest of experimental subjects to investigate the stimuli.
  a. “Social over food preferences scores” can be computed as [((social time)/(social time + food time))*100], where values > 50% indicate that subjects spent more time with the social stimulus, and values < 50% indicate that subjects spent more time with the food stimulus. Alternatively, the inverse can be calculated to determine “food over social preferences scores”, depending on the research question.
    i These scores can be computed for time spent in the social chamber versus the food chamber, time spent in the social investigation zone versus time spent in the food investigation zone, and/or time spent actively investigating the social stimulus versus time spent actively investigating the food stimulus.
  b. Chamber preference can be calculated taking the middle chamber into account, where preference for the social chamber is computed as [(social chamber time/length of test)*100] and preference for the food chamber computed as [(food chamber time/length of test)*100].

## Validation

The Social versus Food Preference Test was validated by examining how acute food deprivation would alter stimulus preference, under the assumption that food deprivation would increase motivation for food and thus bias preference more towards the food stimulus [1,^6^,7]. Experimental subjects were individually housed adult (13–14 week old) Wistar rats (*n* = 7 males, *n* = 5 females) and C57BL/6 mice (*n* = 8 males, *n* = 6 females) [2]. All housing and testing was in accordance with the National Institute of Health *Guidelines for Care and Use of Laboratory Animals* and the Michigan State University Institutional Animal Care and Use Committee (IACUC). Experimental subjects were first habituated to the testing apparatus as described above, and then tested in the Social versus Food Preference Test on two occasions each 48 hrs apart using a within-subjects counterbalanced design (sated × food-deprived). The length of food deprivation was 24 hrs for rats and 18 hrs for mice (per IACUC recommendation and pilot testing to ensure subjects would not lose more than 15% of their body weight). The social stimulus was an unfamiliar age-, sex-, and species-matched conspecific, and each social stimulus was used twice per day in non-successive tests to reduce the number of animals used. The food stimulus was standard laboratory chow (Teklad Irradiated 22/5 Rodent Diet, 8940; ~8 pellets for mice, ~20 pellets for rats). A webcam (Logitech HD Pro-C910) was attached to the ceiling and connected to a PC computer in an adjoining room to record the habituation and test sessions. All other methods were as described above.

Automated behavioral tracking software (AnyMaze, Stoelting) was used to quantify the amount of time experimental subjects spent in each of the three chambers, and the amount of time experimental subjects spent in designated investigation zones around the corrals (rats: head placement within 6 cm of corral edge, mice: center mass placement within 5 cm of corral edge; Fig. 1A). Experimenters, who were unaware of sex and testing conditions, manually scored recorded videos using a freely-available behavioral coding program (Solomon Coder, https://solomon.andraspeter.com/) to quantify the amount of time the experimental subjects spent investigating each of the two stimuli. Social over food preference scores were then computed for all three of these measurements (i.e., chamber, investigation zone, active investigation). There were no significant differences between males and females for any of these behavioral measures in our preliminary analyses [also see: 2], thus data were collapsed across sex. Data were analyzed using mixed-model omnibus ANOVAs [hunger condition (sated, food-deprived; within-subjects factor) × species (rats, mice; between-subjects factor)], and *post hoc* simple effect F-tests were conducted to clarify significant interactions. One-sample t-Tests with a reference value of 50% were used to determine chamber preference during habituation and stimulus preference during tests. Estimates of effect sizes were assessed by partial eta squared (η^2^) or Cohen's d (*d*). All data were analyzed using IBM SPSS Statistics 26, and statistical significance was set at *p* < 0.05.

Neither rats (44.5 ± 3.6, *t*_(11)_ = 1.51, *p* = 0.16) nor mice (54.8 ± 3.93, *t*_(13)_ = 1.22, *p* = 0.24) exhibited a preference for the left chamber versus the right chamber during the habitation session.

**Fig. 2:**
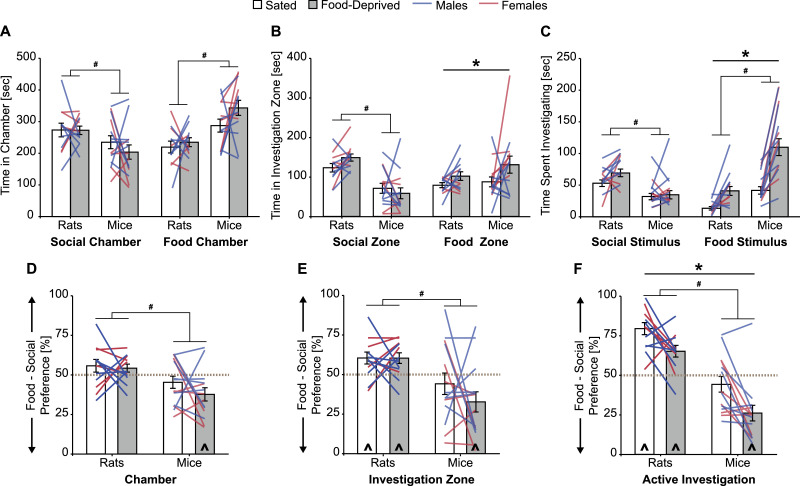
**Food deprivation biases preference more towards the food stimulus in rats and mice**. Food deprivation increased the time spent with the food stimulus (**B** and **C**, right), but did not alter the amount of time spent with the social stimulus (**A–C**, left). Stimulus preference, as measured by automated tracking of chamber time or investigation zone time, was unchanged by food deprivation in rats or mice (**D** and **E**). However, when preference was measured by manually scoring investigation time, food deprivation significantly decreased social over food preference scores (**F**). Specifically, food deprivation attenuated preference for the social stimulus in rats (**F**, left), and produced a preference for the food stimulus in mice (F, right). Rats spent more time with the social stimulus than mice (**A–C**, left) and mice spent more time with food stimulus than rats (**A** and **C**, right), which resulted in higher social over food preference scores in rats compared to mice (**D–F**). Bar graphs display mean ± SEM; * *p* < 0.05, ANOVA main effect of hunger condition; # *p* < 0.05, ANOVA main effect of species; ^ *p* < 0.05, one-sample t-Test from 50% (gray dashed line).

As expected, food deprivation significantly increased the amount of time experimental subjects spent in the investigation zone around the corral containing the food stimulus and the amount of time subjects spent actively investigating the food stimulus, however the amount time spent in the chamber containing the food stimulus was similar between sated and food-deprived conditions (Table 1, Fig. 2A–C). There was a significant hunger condition by species interaction on the time spent investigating the food stimulus (Table 1). *Post hoc* F tests indicated this was because the effect size for the increase in investigation time between sated and food-deprived conditions was larger in mice (*F*_(1, 24)_ = 49.0, *p* < 0.001, *η*^2^ = 0.67) than in rats (*F*_(1, 24)_ = 6.74, *p* = 0.016, η^2^ = 0.22). Mice spent more time than rats in the chamber containing the food stimulus and actively investigating the food stimulus, but time in the investigation zone around the corral containing the food stimulus was similar between rats and mice (Table 1, Fig. 2A–C).

**Table 1:** ANOVA statistics and partial eta squared (*η*^2^) effect sizes; significant effects shown in **bold**.

	*Hunger condition*	*Species*	*Interaction*
Social chamber [s]	*F*_(1,24)_ = 0.62, *p* = 0.48, *η*^2^ = 0.025	***F***_**(1,24)**_ **= 7.23,*****p*** **= 0.013,*****η***^**2**^ **= 0.23**	*F*_(1,24)_ = 0.54, *p* = 0.47, *η*^2^ = 0.022
Food chamber [s]	*F*_(1,24)_ = 2.76, *p* = 0.11, *η*^2^ = 0.10	***F***_**(1,24)**_ **= 22.5,*****p*****< 0.001,*****η***^**2**^ **= 0.48**	*F*_(1,24)_ = 0.90, *p* = 0.35, *η*^2^ = 0.036
Chamber: Social over food preference [%]	*F*_(1,24)_ = 1.32, *p* = 0.26, *η*^2^ = 0.052	***F***_**(1,24)**_ **= 14.3,*****p*****< 0.001,*****η***^**2**^ **= 0.37**	*F*_(1,24)_ = 0.60, *p* = 0.45, *η*^2^ = 0.025
Social zone [s]	*F*_(1,24)_ = 0.36, *p* = 0.55, *η*^2^ = 0.015	***F***_**(1,24)**_ **= 30.1,*****p*****< 0.001,*****η***^**2**^ **= 0.56**	*F*_(1,24)_ = 3.13, *p* = 0.090, *η*^2^ = 0.12
Food zone [s]	***F***_**(1,24)**_ **= 5.34,*****p*** **= 0.048, η**^**2**^ **= 0.15**	*F*_(1,24)_ = 1.90, *p* = 0.18, *η*^2^ = 0.073	*F*_(1,24)_ = 0.41, *p* = 0.53, *η*^2^ = 0.017
Zone: Social over food preference [%]	*F_(_*_1,24)_ = 0.95, *p* = 0.34, *η*^2^ = 0.038	***F***_**(1,24)**_ **= 18.5,*****p*****< 0.001,*****η***^**2**^ **= 0.44**	*F*_(1,24)_ = 0.94, *p* = 0.34, *η*^2^ = 0.038
Social investigation [s]	*F*_(1,24)_ = 3.34, *p* = 0.080, *η*^2^ = 0.12	***F***_**(1,24)**_ **= 20.2*****p*****< 0.001,*****η***^**2**^ **= 0.46**	*F*_(1,24)_ = 1.59, *p* = 0.22, *η*^2^ = 0.062
Food investigation [s]	***F***_**(1,24)**_ **= 44.3,*****p*****< 0.001,*****η***^**2**^ **= 0.65**	***F***_**(1,24)**_ **= 24.3,*****p*****< 0.001,*****η***^**2**^ **= 0.50**	***F***_**(1,24)**_ **= 8.12,*****p*** **= 0.009,*****η***^**2**^ **= 0.25**
Investigation: Social over food preference [%]	***F***_**(1,24)**_ **= 17.2,*****p*****< 0.001,*****η***^**2**^ **= 0.41**	***F***_**(1,24)**_ **= 54.4,*****p*****< 0.001,*****η***^**2**^ **= 0.69**	*F*_(1,24)_ = 0.25, *p* = 0.62, *η*^2^ = 0.01

Across all measurements, the amount of time experimental subjects spent with the social stimulus was similar between sated and food-deprived conditions, but rats spent significantly more time than mice with the social stimulus (Table 1, Fig. 2A–C).

Social over food preference scores as measured by automated tracking of chamber time or investigation zone time were similar between sated and food-deprived conditions (Table 1, Fig. 2D and E). However, when preference was measured by manually scoring investigation time, food deprivation significantly decreased social over food preference scores in rats and mice (Table 1, Fig. 2F). Across all measurements, rats had greater social over food preference scores than mice (Table 1, Fig. 2D–F). Under both sated and food-deprived conditions rats exhibited an equal preference for the social chamber and the food chamber (Fig. 2D), and a significant preference for the social stimulus as measured by investigation zone time or active investigation time (Table 2, Fig. 2E and F). In contrast, for all measurements, mice had no stimulus preference when sated and a food preference when food-deprived (Table 2, Fig. 2D and E).

**Table 2:** One-sample *t*-Test statistics and Cohen's *d* effect sizes;*significant preference for social, ^significant preference for food.

	*Rats*		*Mice*	
	*Sated*	*Food-deprived*	*Sated*	*Food deprived*
Chamber: social over food preference [%]	*t*_(11)_ = 1.31, *p* = 0.22, *d* = 0.38	*t*_(11)_ = 1.48, *p* = 0.17, *d* = 0.43	*t*_(13)_ = 1.33, *p* = 0.21, *d* = 0.36	**^*****t***_**(13)**_ **= 3.05,*****p*** **= 0.009,*****d*** **= 0.82**
Zone: social over food preference [%]	*t_**(11)**_ **= 2.68,*****p*** **= 0.022,*****d*** **= 0.77**	*t_**(11)**_ **= 2.97,*****p*** **= 0.013,*****d*** **= 0.86**	*t*_(13)_ = 0.94, *p* = 0.37, *d* = 0.25	**^*****t***_**(13)**_ **= 2.79,*****p*** **= 0.015,*****d*** **= 0.75**
Investigation: social over food preference [%]	*t_**(11)**_ **= 7.57,*****p*****< 0.001,*****d*** **= 2.19**	*t_**(11)**_ **= 4.00,*****p*** **= 0.022,*****d*** **= 1.16**	*t*_(13)_ = 1.26, *p* = 0.23, *d* = 0.34	**^*****t***_**(13)**_ **= 4.92,*****p*****< 0.001,*****d*** **= 1.31**

To summarize, while there were robust differences between Wistar rats and C57BL/6 mice in stimulus investigation patterns, how their investigation patterns changed in response to the food deprivation manipulation was similar. Specifically, food deprivation increased the time spent with the food stimulus, and this decreased social over food preference scores. Importantly, the data presented here illustrate the benefits of conducting manual scoring for active investigation time, since this measurement typically had larger effect sizes than those observed for automated tracking of chamber time or investigation zone time.

## Additional information

### Adjustments to reduce climbing of the corrals and/or escape from the apparatus

**Fig. 3:**
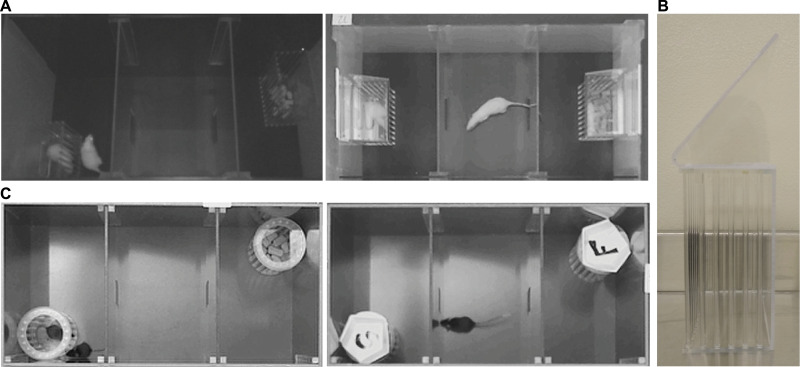
In initial testing, rats (**A**, left) and mice (**C**, left) would climb corrals and/or escape the apparatus. Altering corral placement and adding a wedge top to the corrals prevented climbing and escape in rats (**A**, right; **B**), and the addition of a weigh boat to the top of the corrals prevented climbing and escape in mice (C, right; marked to indicate stimulus inside).

A common early issue in developing the Social versus Food Preference Test was that some animals would climb up the corrals and sit on the top. From the top of the corrals some animals were then able to jump out of their testing arena and escape completely or jump into the neighboring testing arena and disrupt the testing of other subjects. For rats, these issues were mitigated by placing corrals in the center of the back wall instead of in a corner (Fig. 3A). To further reduce climbing and/or subsequent escape, the corrals were modified so that the tops were no longer flat. For the rat corrals this was achieved by adding a Plexiglas wedge to the lid of the corral (Fig. 3A and B), and for the mouse corrals this was achieved by taping a weigh boat to the top of the corral (Fig. 3C). These changes greatly decreased the frequency of climbing and escape, and in subsequent testing nearly all subjects spent the entire 10 min of each test exploring the apparatus and investigating the stimuli. Any corral design or modification that eliminates a flat top would be recommended. If using a pencil cup holder for mice, placing a second weighted cup on top is a commonly used alternative solution [5].

### Rats prefer a food stimulus over no stimulus

**Fig. 4:**
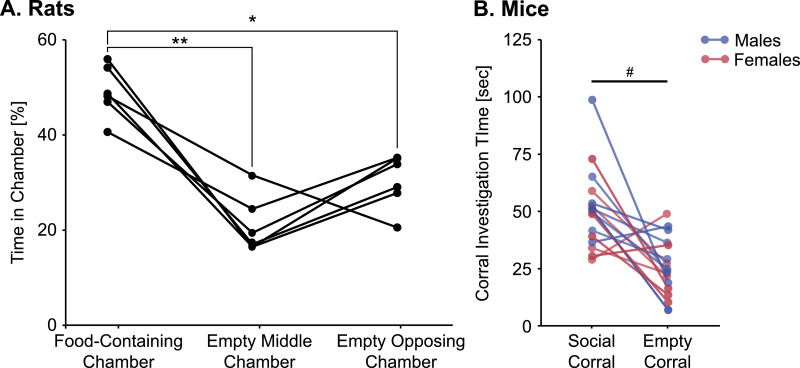
Male Wistar rats spent more time in the chamber containing food pellets compared to either other empty chamber (**A**). Male and female C57BL/6 mice spent more time investigating a corral containing a social stimulus compared to an empty corral (**B**); *p < 0.05, **p <0.001, Bonferroni *post hoc* paired comparisons; # *p* = 0.001, paired t-Test.

To determine if a food preference could be induced in rats, individually housed 9–10 week old male Wistar rats (*n* = 6) were first habituated to the apparatus and then tested the following day following acute (24 h) food deprivation. During the test, subjects were placed into the center chamber and allowed to freely explore for 10 min. In one end chamber was a glass staining dish (107 × 87 × 70 mm) containing 3 chow pellets available for consumption, and the middle and opposing chambers were empty (i.e., did not contain corrals or dishes). Pellets were replaced, and the dish cleaned between subjects. Behavior was video recorded, and later manually scored using Solomon Coder for the percent of time subjects spent in each chamber. A within-subjects ANOVA showed a significant effect of chamber (*F*_(2, 10)_ = 25.0, *p* < 0.001), and Bonferroni *post hoc* paired comparisons confirmed that rats spent a greater percent of time in the chamber containing the food compared to the percent of time spent in the opposing (*p* = 0.013) or middle chambers (*p* = 0.003; Fig. 4A). The percent of time spent in the opposing and middle chambers was similar (*p* = 0.23). This suggests that in the absence of a social stimulus, rats can exhibit a preference for a food stimulus.

### Mice prefer a social stimulus over no stimulus

To determine if a social preference could be induced in mice, pair-housed and *ad lib* fed 6–14 week old C57BL/6 mice (*n* = 8 males, *n* = 8 females) were first habituated to the apparatus and then tested the following day [2]. During the test, subjects were placed into the center chamber and allowed to freely explore for 10 min. In one end chamber was a corral containing an unfamiliar age- and sex-matched conspecific, and in the other end chamber was an empty corral. Behavior was video recorded, and later manually scored using Solomon Coder for the amount time subjects spent investigating each corral. A paired samples t-Test showed that mice spent significantly more time investigating the corral containing the social stimulus compared to the empty corral (*F*_(1,15)_ = 3.88, *p* = 0.001; Fig. 4B). This suggests that in the absence of a food stimulus, mice can exhibit a preference for a social stimulus.

### Using a freely-accessible food stimulus versus an inaccessible corralled food stimulus

**Fig. 5:**
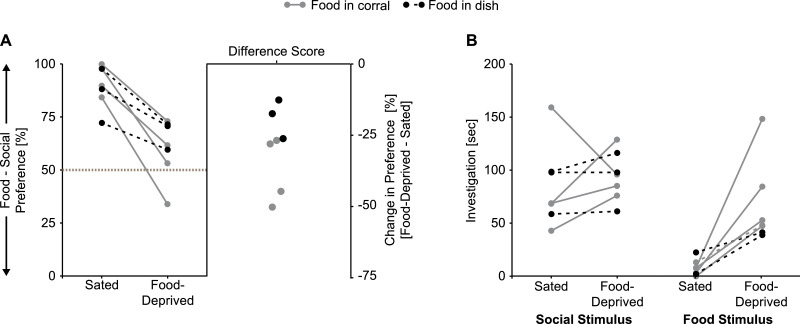
Male Wistar rats that were allowed free access to the food pellets showed less of a food deprivation-induced reduction in social over food preference (**A**) and spent less time investigating the food stimulus (**B**) than subjects for whom the food was inaccessible due to the food being placed in a corral.

In a two-chamber social interaction assay assessing the effects of hunger signals on social interest in mice, a food pellet was secured to the apparatus with adhesive putty which allowed for food consumption during the test [1]. In an initial pilot experiment, we examined whether stimulus investigation patterns would be similar when the food stimulus was freely-accessible (by placing food pellets in a glass staining dish; 107 × 87 × 70 mm) or inaccessible (by placing food pellets in corrals identical to those used for the social stimuli). Individually housed 5–6 week old male Wistar rats were first habituated to the testing apparatus and then exposed to the Social versus Food Preference Test on two occasions 48 hrs apart using a within-subjects counter-balanced design [sated × acutely food-deprived for 24 hrs]. For all subjects, the social stimulus was an unfamiliar age- and sex-matched conspecific, and the food stimulus was standard laboratory chow (Teklad Irradiated 22/5 Rodent Diet, 8940). For half of the subjects (*n* = 3) 3 chow pellets were placed into a rectangular glass staining dish allowing for consumption, and for the other half of the subjects (*n* = 4) 3 chow pellets were placed into a corral. Behavior was video recorded, and later manually scored using Solomon Coder for the amount of time subjects spent investigating each stimulus. All other methods were as described in **Method details**. This pilot was underpowered for statistical analyses, however, the average food deprivation-induced decrease in social over food preference was lower in subjects that had free-access to the food compared to subjects whose food was inaccessible (Fig. 5A), and this was driven by average lower levels of food stimulus investigation in subjects that had free-access to the food compared to subjects whose food was inaccessible (Fig. 5B). Additionally, one subject in the free-access condition carried a food pellet from the dish to the chamber with the social stimulus complicating the coding of behavior. Further, the expression of motivated behaviors is classically broken down into appetitive (i.e., initial, seeking) and consummatory (i.e., final, satisfaction of drive) behaviors [8], and corralling the food narrows the behavioral analyses to appetitive behaviors for both stimuli. For all these reasons, placing food in the corrals is recommended over free-access.

### Monitoring post-test food consumption for 30 min versus 1 h

**Fig. 6:**
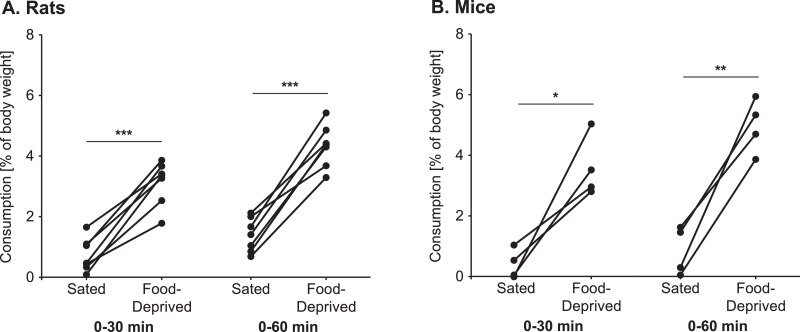
Monitoring post-test food consumption for 30 min is sufficient to observe differences between sated and food-deprived conditions in male Wistar rats (**A**) and female C57BL/6 mice (**B**); *p < 0.05, **p < 0.01, ***p < 0.001, Bonferroni *post hoc* paired comparisons.

For experiments where subjects were food-deprived, we wanted to behaviorally assess hunger by examining food consumption in the homecage immediately following exposure to the Social versus Food Preference Test. Initial pilot tests were conducted to determine the length of time these post-test food consumption must be in order to observe differences in consumption between sated and food-deprived conditions. Specifically, individually housed male Wistar rats (*n* = 7, 5–6 weeks old) and female C57BL/6 mice (*n* = 4, 5–6 weeks old) were first habituated to the testing apparatus and then exposed to the Social versus Food Preference Test on two occasions 48 hrs apart using a within-subjects counter-balanced design [sated × acutely food-deprived (rats: 24 h, mice: 18 h)]. For all subjects, the social stimulus was an unfamiliar age-, sex-, and species-matched conspecific, and the food stimulus was standard laboratory chow (Teklad Irradiated 22/5 Rodent Diet, 8940). Immediately following each test, subjects were weighed and body weights recorded. Subjects were then returned to their homecage where they were given a pre-weighed amount of food pellets which was replaced and weighed after 30 min and 60 min. All other methods were as described in **Method details***.* Consumption was computed as percent of body weight [(grams consumed/body weight in grams)*100], and analyzed using two-way within-subjects ANOVAs [hunger condition (sated, food-deprived) × time point (30 min, 60 min)]. Bonferroni *post hoc* planned comparisons were used to assess the effect of hunger condition at each time point. In rats, there were main effects of both hunger (*F*_(1,6)_ = 199, *p* < 0.001) and time point (*F*_(1,6)_ = 42.6, *p* < 0.001); *post hoc* tests confirmed there was a difference between the sated and food-deprived conditions at both the 30 min and 60 min time points (*p* < 0.001, both; Fig. 6A). In mice, there was a main effect of hunger (*F*_(1,3)_ = 35.1, *p* = 0.01) and a hunger by time point interaction (*F*_(1,3)_ = 10.8, *p* = 0.046); *post hoc* tests confirmed there was a difference between the sated and food-deprived conditions at both the 30 min (*p* = 0.02) and 60 min time points (*p* = 0.005; Fig. 6B). Thus, monitoring post-test consumption for 30 min is sufficient to observe the effects of the acute food deprivation.

### Testing during the dark phase versus the light phase

**Fig. 7:**
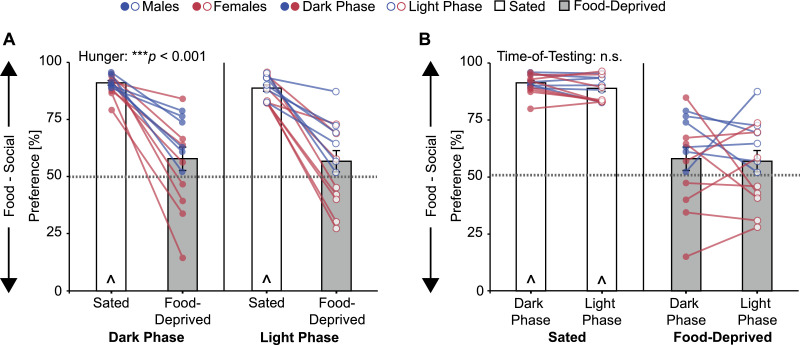
Food deprivation abolished preference for the social stimulus in adolescent Wistar rats (**A**), while time-of-testing had no effect of stimulus preference (**B**; same data re-plotted to highlight main effects). Bar graphs (mean ± SEM) and statistics collapsed across sex; *** *p <* 0.001 repeated measures ANOVA; ^ *p* < 0.05, one-sample *t*-Test from 50% (gray dashed line); n.s. = not significant.

To determine whether stimulus preference would be affected by the time-of-testing, we tested how subjects’ preferences to investigate the social stimulus (novel age- and sex- matched conspecific) versus the food stimulus (standard laboratory chow) were modulated by acute 24 hr food deprivation and the time-of-testing. Specifically, we compared behavior during the start of the dark phase (zeitgeber time 12) to behavior during the middle of the light phase (zeitgeber time 7). Individually housed adolescent Wistar rats (*n* = 6 males, *n* = 8 females; 5–6 weeks old) were first habituated to the testing apparatus and then tested on four occasions each 48 hrs apart using a within-subjects 2 × 2 counterbalanced design [sated/food-deprived × dark phase/light phase]. Behavior was video recorded, and later manually scored using Solomon Coder for the amount time subjects spent investigating each stimulus. A repeated measures ANOVA [hunger condition (sated, food-deprived) × time-of-testing (dark phase, light phase)] showed a significant main effect of hunger condition (*F*_(1,13)_ = 69.1, *p* < 0.001; Fig. 7A), but no main effect of (*F*_(1,13)_ = 0.47, *p* = 0.51; Fig. 7B) or interaction with (*F*_(1,13)_ = 0.065, *p* = 0.80) time-of-testing on stimulus preference. One-sample t-Tests with a reference value of 50% showed that adolescent Wistar rats had a significant social preference under sated conditions (dark phase: *t*_(13)_ = 38.8, *p* < 0.001; light phase: *t*_(13)_ = 27.4, *p* < 0.001) and no stimulus preference under food-deprived conditions (dark phase: *t*_(13)_ = 1.53, *p* = 0.15; light phase: *t*_(13)_ = 1.44, *p* = 0.17; Fig. 7). These results support the use of the Social versus Food Preference Test during the light phase which can alleviate practical difficulties associated with dark phase testing [9].

### Custom-construction allows for a modular design that can be utilized in additional behavioral assays

**Fig. 8:**
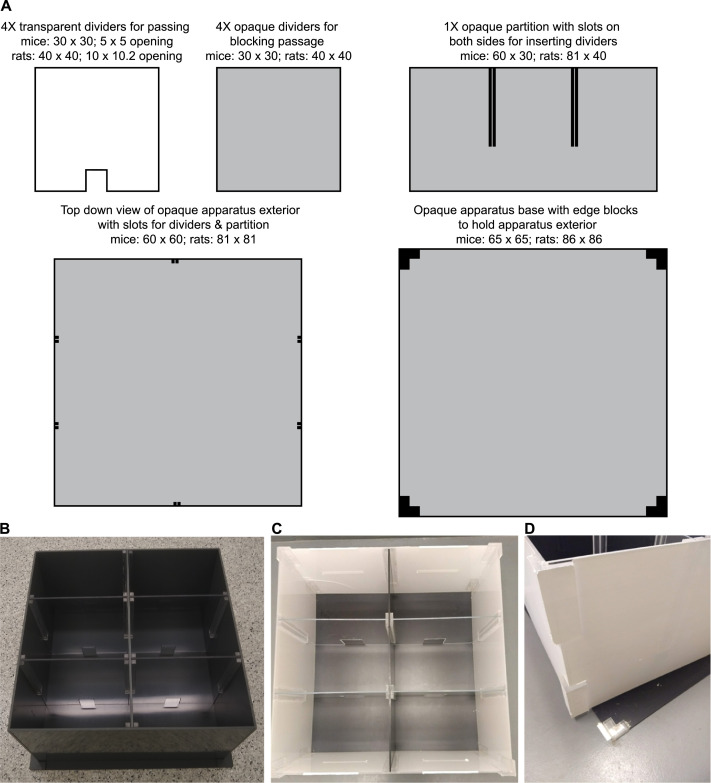
Dimensions, in cm, for the custom construction of a modular testing apparatus (**A**). A modular design allows for diverse uses: a single large open field arena, multiple smaller open field arenas of various sizes, or 2 two- or three-chamber arenas (mice: **B**, rats: **C**), and facilities easy cleaning of the dividers, partition, and base (D, exterior of apparatus lifted off base during cleaning).

While three-chamber testing apparatuses are commercially available, we had both our rat-sized and mouse-sized apparatuses custom-constructed at a much lower cost. For example, the cost for the custom-construction of our modular mouse-sized testing apparatus and the accompanying corrals by our campus Machine Shop (labor + materials) was 3–4 times cheaper than purchasing commercially available options. In addition to being budget-friendly, custom-construction allowed us to create a modular design which maximizes limited space and facilitates easy cleaning (Fig. 8). The apparatus starts as a large, square arena which can be used for open-field tests to assess anxiety and general locomotor activity [10], for novel object recognition tests to assess learning and memory [11], or a variety of other behavioral tests. The insertion of an opaque partition divides the apparatus into two separate parts, and the insertion of transparent or opaque dividers can further divide the space to meet experimental needs. For the Social versus Food Preference Test we utilized two transparent dividers in each half of the apparatus in order to create two three-chamber testing arenas, and thus were able to run two subjects at the same time and record both from a single overhead camera. The apparatus could also be divided into a variety of smaller open-field arenas or two-chamber arenas. With some removable Con-Tact paper, it would be possible to create a modified conditioned place preference setup [12] to assess learned place preferences as opposed to the real-time place preferences assessed in assays like the Social versus Food Preference Test. Cleaning is simple since all parts can be easily inserted and removed, including the main apparatus from the base, which also prevents the build-up of debris that can occur in edges/corners.

## Declaration of Competing Interest

The authors declare that they have no known competing financial interests or personal relationships that could have appeared to influence the work reported in this paper.

## References

[1] Burnett C.J., Li C., Webber E., Tsaousidou E., Xue S.Y., Bruning J.C., Krashes M.J. (2016). Hunger-driven motivational state competition. Neuron.

[2] Reppucci C.J., Brown L.A., Chambers A.Q., Veenema A.H. (2020). Wistar rats and C57BL/6 mice differ in their motivation to seek social interaction versus food in the social versus food preference test. Physiol. Behav..

[3] Smith C.J., Wilkins K.B., Mogavero J.N., Veenema A.H. (2015). Social novelty investigation in the juvenile rat: modulation by the mu-opioid system. J. Neuroendocrinol..

[4] DiBenedictis B.T., Cheung H.K., Nussbaum E.R., Veenema A.H. (2020). Involvement of ventral pallidal vasopressin in the sex-specific regulation of sociosexual motivation in rats. Psychoneuroendocrinology..

[5] Moy S.S., Nadler J.J., Perez A., Barbaro R.P., Johns J.M., Magnuson T.R., Crawley J.N. (2004). Sociability and preference for social novelty in five inbred strains: an approach to assess autistic-like behavior in mice. Genes Brain Behav..

[6] Burnett C.J., Funderburk S.C., Navarrete J., Sabol A., Liang-Guallpa J., Desrochers T.M., Krashes M.J. (2019). Need-based prioritization of behavior. Elife.

[7] Martin L., Sample H., Gregg M., Wood C. (2014). Validation of operant social motivation paradigms using BTBR T+tf/J and C57BL/6J inbred mouse strains. Brain Behav.

[8] Craig W. (1918). Appetites and aversions as constituents of instincts. Biol. Bull..

[9] Yang M., Weber M.D., Crawley J.N. (2008). Light phase testing of social behaviors: not a problem. Front. Neurosci..

[10] Gould T.D., Dao D.T., Kovacsics C.E., Gould T.D. (2009). The open field test. Mood and Anxiety Related Phenotypes in Mice.

[11] Antunes M., Biala G. (2012). The novel object recognition memory: neurobiology, test procedure, and its modifications. Cogn. Process..

[12] Prus A.J., James J.R., Rosecrans J.A., Buccafusco J.J. (2009). Conditioned place preference. Methods of Behavior Analysis in Neuroscience.

